# Complete prevention of HTLV-1 infection in humanized mice (hu-PBL SCID) by a neutralizing monoclonal antibody to envelope gp46

**DOI:** 10.1186/1742-4690-11-S1-O9

**Published:** 2014-01-07

**Authors:** Mineki Saito, Reiko Tanaka, Akira Kodama, Yuetsu Tanaka

**Affiliations:** 1 Department of Immunology, Graduate School of Medicine, University of the Ryukyus, Okinawa, Japan; 2 Department of Microbiology, Kawasaki Medical School, Kurashiki, Japan

## 

Human T-cell leukemia virus type 1 (HTLV-1) causes both neoplastic and inflammatory diseases: adult T-cell leukemia (ATL) and HTLV-1-associated myelopathy/tropical spastic paraparesis (HAM/TSP). Since these disabling and/or life threatening diseases are not yet curable, it is important to prevent new infections. In this study, we have established a simple humanized mouse model of HTLV-1 infection for evaluating therapeutic and immunomodulatory interventions. Using this model, we tested the effect of HTLV-1 specific neutralizing antibodies. HTLV-1-negative normal human peripheral blood mononuclear cells (PBMCs) were transplanted directly into the spleens of severely immunodeficient mice (NOD/SCID/γCnull: NOG) together with the mitomycin-treated HTLV-1 producing T cells (ILT-M1). Before (one hour) and after (24 hours) transplantation of human PBMCs, monoclonal antibodies against HTLV-1 as well as human IgG isolated from both HTLV-1 infected and non-infected individuals were inoculated intraperitonealy. On day 14, human PBMCs were isolated from mouse spleen, and tested for HTLV-1 infection by real time PCR and flow cytometry. Similar to the naturally HTLV-1 infected PBMCs, both CD4+ and CD8+ T cells isolated from untreated or isotype antibody treated mice were found to be HTLV-1 infected, and the CD8+ T cells harbored HTLV-1 to a lesser extent. Also, HTLV-1 Tax expression was negative in isolated human PBMCs but became positive after 16 hours of culture. Although non-neutralizing monoclonal antibodies to gp46, monoclonal antibody to gag p19, and normal human IgG did not block the infection, neutralizing monoclonal antibody to gp46 and human anti-HTLV-1 IgG completely blocked the infection. Our findings provide a new strategy for preventing initial HTLV-1 infection and blocking further spread in vivo. The potential mechanisms involved in the antibody effect will also be discussed.

